# Atomic structure of the Se-passivated GaAs(001) surface revisited

**DOI:** 10.1038/s41598-023-45142-y

**Published:** 2023-10-24

**Authors:** Akihiro Ohtake, Takayuki Suga, Shunji Goto, Daisuke Nakagawa, Jun Nakamura

**Affiliations:** 1 National Institute for Materials Science (NIMS), Tsukuba, 305-0044 Japan; 2 Department of Engineering Science, The University of Electro-Communications (UEC-Tokyo), Chofu, Tokyo 182-8585 Japan

**Keywords:** Surfaces, interfaces and thin films, Nanoscale materials

## Abstract

We present a combined experimental and theoretical study of the Se-treated GaAs(001)-($2\times 1$) surface. The ($2\times 1$) structure with the two-fold coordinated Se atom at the outermost layer and the three-fold coordinated Se atom at the third layer was found to be energetically stable and agrees well with the experimental data from scanning tunneling microscopy, low energy electron diffraction, and x-ray photoelectron spectroscopy. This atomic geometry accounts for the improved stability of the Se-treated surface against the oxidation. The present result allows us to address a long-standing question on the structure of the Se-passivated GaAs surface, and will leads us to a more complete understanding of the physical origin of the electrical and chemical passivation of Se-treated GaAs surface.

## Introduction

The passivation of III-V semiconductor surface by group VI elements of S and Se is known to be effective in improving the electrical properties of devices^1–4^. While the S/Se passivated surfaces have been extensively studied, especially, in the early 1990s, the mechanism is still far from being well understood. The structure identification of the S/Se-passivated GaAs surface is a key to understand the mechanism of the surface modification induced by the passivation.

In this paper, we present a systematic study on the atomic structure of the Se-treated GaAs(001) surface. Early studies have shown that the GaAs(001) surface treated by Se shows a ($2\times 1$) reconstruction^5–11^, and a large number of structure models has been proposed for the Se-induced ($2\times 1$) structure^6–9^. In the most of the proposed structure models, Se atoms are located on the GaAs surface and are also incorporated into the subsurface layers^5–13^. It has been also suggested that $\hbox {Ga}_{2}\hbox {Se}_{3}$-like surface layers are formed on the Se-treated GaAs surface^5–10^.

**Figure 1:**
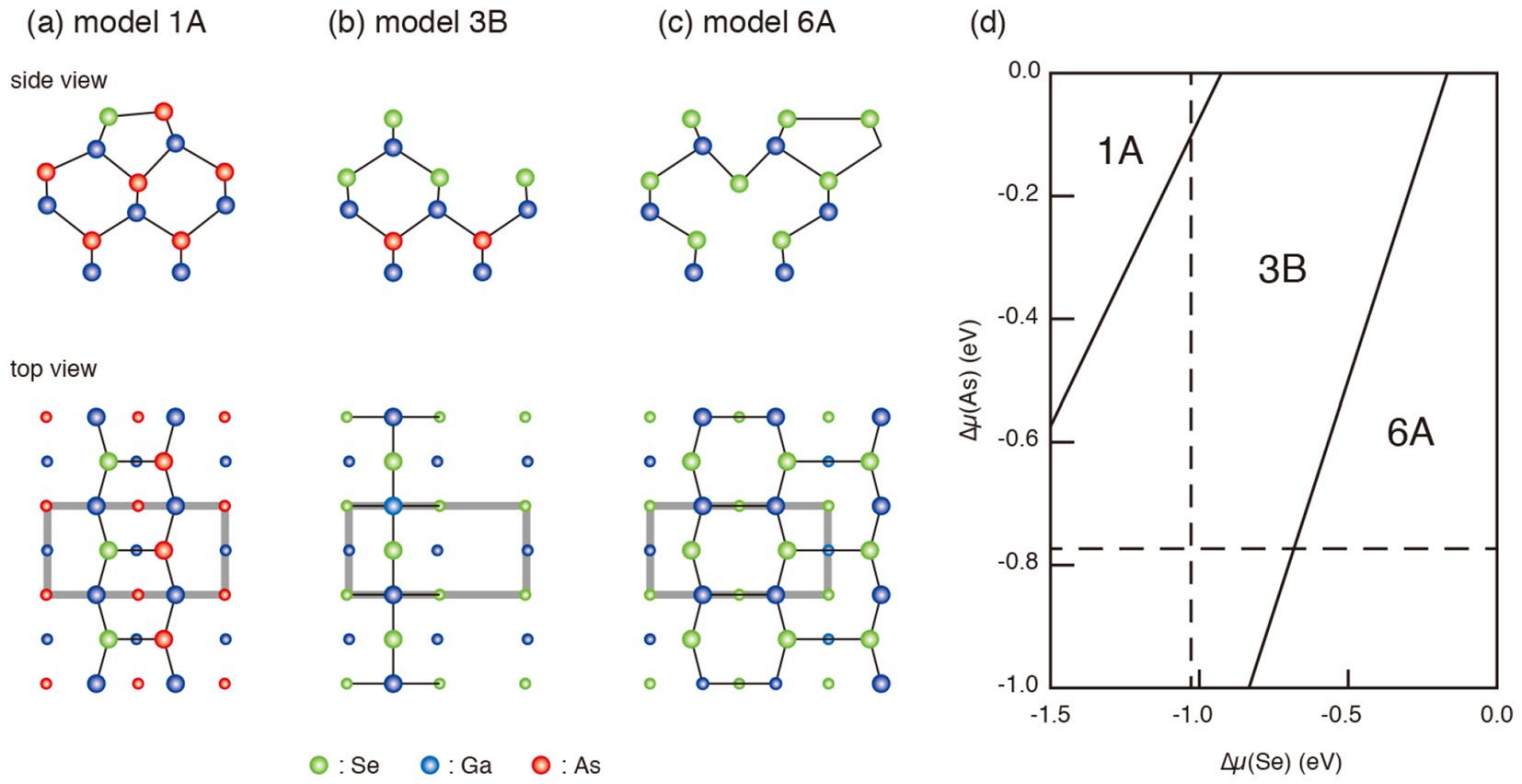
Possible structure models for the GaAs(001)-($2\times 1$)-Se surface (**a**)–(**c**). **d** Phase diagram of the GaAs(001)-($2\times 1$)-Se structures as functions of the relative potentials of As and Se with respect to their bulk phases. Vertical (horizontal) dashed line shows the chemical potential of Se (As) for the bulk $\hbox {Ga}_{2}\hbox {Se}_{3}$ (GaAs).

Using first-principles calculations, Gundel and Faschinger^14^ have studied 11 structure models that satisfy the electron counting rule^15^ and have found that three structure models shown in Fig. 1a–c are stable^14^. The 1A model has the Se-As dimer at the outermost layer, while the 6A model consists of the surface Se-Se dimer and Ga vacancies at the fourth atomic layer. The existence of surface dimers in the 1A and 6A models is in good agreement with the models proposed in refs.^6,8,9^. On the other hand, in the 3B model, the Se atom is located at the bridge site and is bound with two Ga atoms at the second layer. While the relative stability of the three structures strongly depends on the surface composition^14^, only the 3B model could account for the experimental data from scanning tunneling microscopy/spectroscopy (STM/STS) and photoemission spectroscopy^16,17^.

Most of structure models proposed so far, including the models shown in Fig. 1a–c, have been based on the experimental data from STM and photoemission spectroscopy measurements. Thus, the structure identification of the Se-treated GaAs surface is not fully convincing: it is difficult to obtain detailed structure information, such as atomic position, atom types, and bond length, from these experimental techniques. Here, we present the quantitative low energy electron diffraction (LEED) analysis for the Se-treated GaAs(001)-($2\times 1$) surface. We found that there exist only one intrinsic ($2\times 1$) structure at least under the present experimental condition: the ($2\times 1$) surface has the 0.5 ML of Se at the outermost layer and 1.0 ML Se at the third atomic layer (3B model in Fig. 1b). The structure model accounts for the experimental results from LEED, STM and x-ray photoelectron spectroscopy (XPS), and is found to be energetically stable. In addition, we found that the atomic geometry effectively suppresses the formation of As oxides, providing a mechanism for the reduction of surface-state density.

## Methods

### Experiments

**Figure 2:**
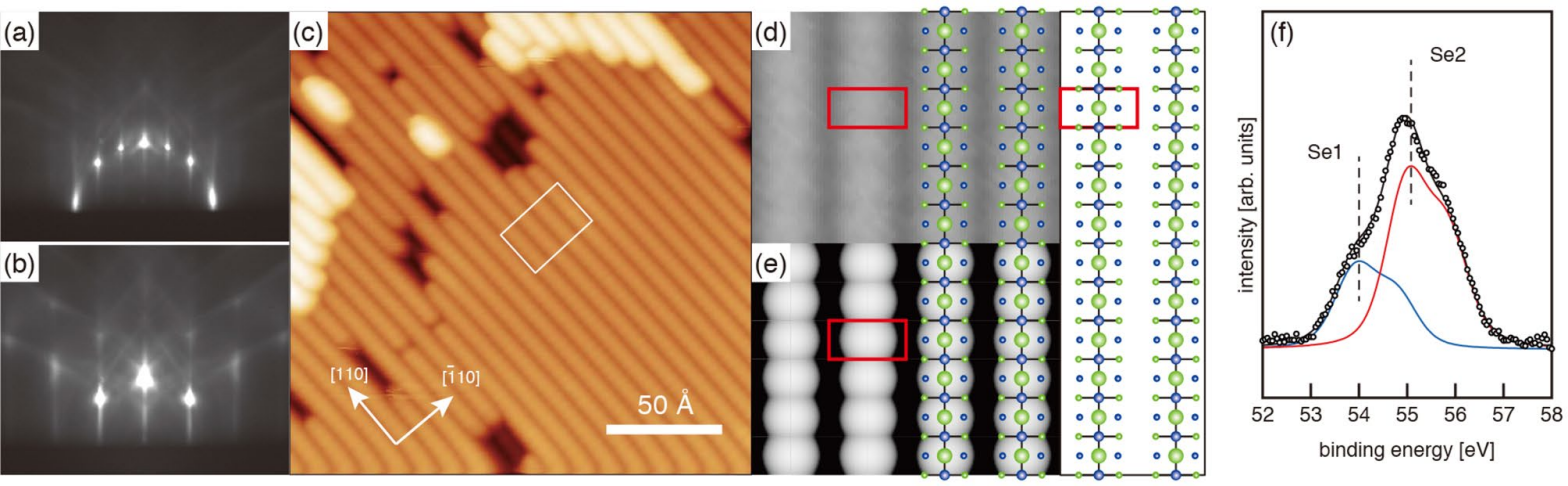
RHEED patterns of the Se-treated GaAs(001)-($2\times 1$) surface taken along the [110] (**a**) and [${\overline{1}}$10] (**b**) directions. (**c**) Typical filled-state STM image of the GaAs(001)-($2\times 1$)-Se surface. The image was taken with a sample bias of $-3$ V. **d** Magnified STM image. **e** Simulated STM image of the 3B model using a filled-state bias of 3V. **f** Se 3d photoelectron spectrum measured from the Se-treated ($2\times 1$) surface.

The samples were prepared in a multi-chamber ultra-high vacuum (UHV) system consisting of molecular beam epitaxy chambers for the growth of GaAs and for the Se treatment^18,19^. The system is equipped with STM and XPS apparatuses for on-line characterization. The clean GaAs(001)-($2 \times 4$) surfaces were obtained by growing an undoped layer (0.5 $\mu$m) on a thermally cleaned Si-doped GaAs(001) substrate. The clean GaAs samples were transferred to another UHV chamber via UHV transfer modules (< $2\times 10^{-9}$ Torr) for the Se treatments. The beam equivalent pressure of Se is controlled to $5 \times 10^{-9}$ Torr. The Se-treatment processes were monitored by in-situ reflection high-energy electron diffraction (RHEED) with electron beam energy of 15 keV. When the GaAs(001)-($2 \times 4$) surface was exposed to the Se molecular beam at $300^{\circ }\hbox {C}$, a diffuse ($2\times 1$) RHEED patterns were observed. After being annealed at $600^{\circ }\hbox {C}$ under the Se flux, the sample showed the sharp ($2\times 1$) RHEED pattern (Fig. 2a and b). Then the substrate temperature is decreased and the Se shutter was closed at $300^{\circ }\hbox {C}$. In the present experiments, we prepared the Se-treated surfaces also on the more Ga-rich ($4 \times 6$)^20^ and As-rich phases of *c*($4 \times 4$)$\alpha$ and *c*($4 \times 4$)$\beta$ surfaces^21^ and confirmed that the initial surface reconstruction hardly affect the structure of the Se-treated ($2\times 1$) surface.

The Se-treated ($2\times 1$) surface was analyzed by LEED (OCI LEED 600), STM (Omicron Micro STM), and XPS (Surface Science Instrument M-Probe). The LEED patterns at room temperature were acquired with a 1 eV step in the energy range of $30-380$ eV. The LEED intensity-voltage ($I-V$) curves for 11 non-equivalent beams (7 integral- and 4 fractional-order beams) were extracted from LEED patterns with the background being subtracted. The total cumulative energy range was approximately 3190 eV (1976 and 1214 eV for integer- and fractional- order beams, respectively).

All the STM images were collected at room temperature in the constant current mode with a tunneling current of 0.1 nA and a sample voltage of $-3$ V. XPS measurements were performed using monochromatic Al K$\alpha$ radiation (1486.6 eV). Photoelectrons were detected at an angle of 35$^{\circ }$ from the surface. The Se 3d, As 3d, and Ga 3d spectra were measured and fitted using a Voigt function with the ratio of Gaussian to Lorentzian components fixed at 2.5. Peak separations of 0.85 eV, 0.68 eV, and 0.45 eV are assumed for the 5/2 and 3/2 spin-orbit components of Se 3d, As 3d, and Ga 3d, respectively.

### Calculations

We performed first-principles calculations^22,23^ within the DFT^24^ in the generalized gradient approximation^25^. The potentials are described by ultrasoft pseudopotentials in the Vanderbilt form^22^. The valence electron configurations are 4$\hbox {s}^{2}$4$\hbox {p}^{1}$ for Ga, 4$\hbox {s}^{2}$4$\hbox {p}^{3}$ for As, and 4$\hbox {s}^{2}$4$\hbox {p}^{4}$ for Se. The calculated lattice constant of GaAs is 5.734 Å, which is close to the experimental value of 5.6538 Å. A slab geometry was used for the simple calculation, which has the supercell consisting of 10 atomic layers and of vacuum region (20 Å in thickness). The back side of the slab is terminated with fictitious H atoms, which eliminate artificial dangling bonds and prevent it from coupling with the front side. The wave functions were expanded by the plane wave basis set with a cutoff energy of 36 Ry. $4 \times 8\times 1$ *k* points were used for the integration in *k* space in the Brillouin zone for the ($2\times 1$) unit cell.

## Results and discussion

We first carried out the DFT calculations for several possible structure models to examine the relative stability. Figure 1a–c show structure models proposed for the GaAs(001)-($2\times 1$)-Se surface. Because of the different numbers of Se and As atoms per unit cell, we have to take into account the chemical potentials of Se [${\Delta }\mu$(Se)] and As [${\Delta }\mu$(As)] to compare the total energies for different models. The phase diagram in dependence upon ${\Delta }\mu$(Se) and ${\Delta }\mu$(As) is shown in Fig. 1d. While the 1A model is the most stable at lower and higher limits of ${\Delta }\mu$(Se) and ${\Delta }\mu$(As), respectively, the 6A model becomes energetically favorable for higher ${\Delta }\mu$(Se) and lower ${\Delta }\mu$(Se). The 3B model is the most stable between the two regions. These results are in good agreement with earlier DFT results^14^.

Figure 2c shows a typical filled-state STM image of the Se-treated GaAs(001)-($2\times 1$) surface. Bright lines running along the [110] direction are separated by dark rows with a spacing of 8 Å (corresponding to the 2$\times$ periodicity). Such a feature has been also reported in earlier papers^8,9,11^. In the magnified image (Fig. 2d), a single bright feature per ($2\times 1$) unit cell is observed. This feature is reproduced in the simulated image (Fig. 2e) extracted using the Tersoff-Hamann formalism^26^ for the 3B model. On the other hand, simulated images for both 1A and 6A models show two bright features per unit cell (see Figure S1 in the Supplementary Material). Thus, on the basis of the simple interpretation of STM images, the 3B model is most probable among the three models shown in Fig. 1a–c, as discussed in ref.^16^.

**Figure 3:**
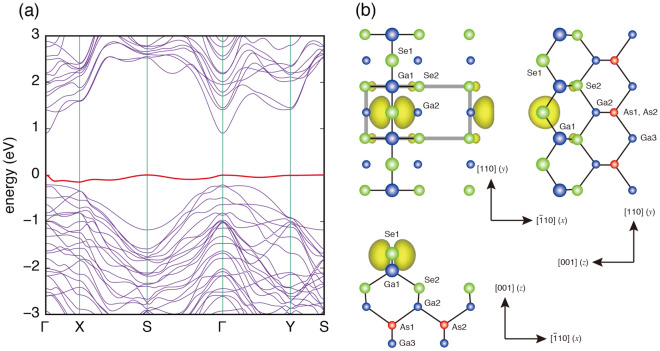
**a** Energy band structure of the 3B model. The energy of the top of the valence band is set to be 0 eV. **b** The probability density of the wave function for the flat band (red line in (**a**)) at the S point.

Here, we examined the electronic structure of the 3B structure in detail in order to investigate the origin of the “cocoon-shaped” bright spots obtained by STM. Figure 3a shows the electronic band structure for the 3B model. As indicated by the red line, the valence band edge forms an extremely flat band. To investigate the origin of this flat band, the probability density of the wave function for this band at the S point is examined as shown in Fig. 3b. It is clearly seen that the state is associated with the fully-occupied, localized $p_{x}$ orbital on the topmost Se1 atom, not with the dangling bond. The bright spots observed in Fig. 2c and d, extending in the [${\overline{1}}$10] (*x*) direction at the topmost surface, have been shown to originate from fully-occupied $p_{x}$ orbitals on the surface, which are not engaged in bonding with any surrounding atoms.

The unique feature of the 3B structure is the existence of the two-fold coordinated Se1 atom. The stability of the structure is closely related with the localization of fully occupied $p_{x}$ orbitals to Se1. In addition to this orbital, there is a lone pair just above the Se1 atom. As a result, the Se1 atom has two remaining electrons which are consumed to form the bonds with two second-layer Ga atoms. Thus, according to the electron counting model^15^, the Se1 atom at the outermost layer lacks 1/2 electrons per ($2\times 1$). On the other hand, the three-fold coordinated Se2 atom located at the third layer has excess 1/4 electrons. Consequently, the 3B structure is electronically stabilized by transferring 1/$4 \times 2$ electrons from two Se2 atoms to the Se1 atom.

The validity of the 3B model was further confirmed by the LEED $I-V$ curve analysis on the basis of dynamical diffraction theory. LEED $I-V$ curves were calculated using SATLEED package provided by Barbieri and Van Hove^27,28^. The present calculation used 10 phase shifts for the description of the electron-crystal interaction. The inner potential $V_{0}+iV_{im}$ was set to be independent of energy: the real part $V_{0}$ was initially set to be 10 eV and adjusted during the fitting process and the imaginary part $V_{im}$ was set to be $-4$ eV. The isotropic thermal vibrational amplitudes represented by Debye temperatures were also optimized to obtain good agreement with the experimental $I-V$ curves. The resultant Debye temperatures for the surface atoms are 160K (Se1), 230K (Ga1), and 220K (Se2) and those for bulk GaAs are 300K (Ga) and 310K (As). To quantify the agreement between measured and calculated $I-V$ curves, we use Pendry’s reliability factor $R_{\textrm{P}}$^29^.

**Figure 4:**
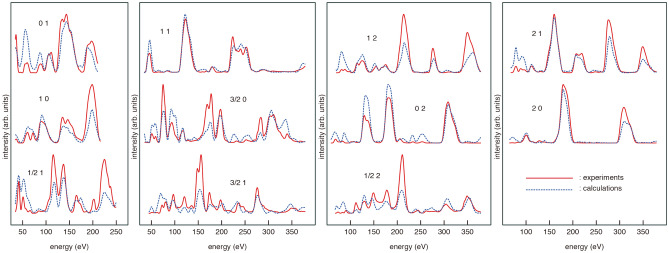
Experimental (solid lines) LEED $I-V$ curves measured from the Se-treated GaAs(001)-($2\times 1$) surface at room temperature. Dashed lines show $I-V$ curves calculated for the 3B model using the atomic coordinates obtained from the DFT calculations.

Figure 4 shows measured LEED $I-V$ curve together with calculated ones for the 3B model. The structure parameters of the 3B models (Fig. 3b) obtained from the DFT calculations are listed in Table 1. This structure yields the R factor of $R_{\textrm{P}}=0.31$, showing a good agreement with the LEED experiments. When the structural parameters were optimized, the agreement was slightly improved ($R_{\textrm{P}}=0.28$). The LEED analysis was carried out also for other structure models of 1A and 6A. These models gave *R* factors larger than 0.6 even after the structure optimization. From these LEED results, it is most likely that the Se-treated GaAs surface has the 3B structure.

The structure parameters in the optimized model (Fig. 3b) are listed in Table I: the atomic coordinates obtained from the LEED analysis agree well with those from the DFT calculations: deviations in absolute coordinates are less than 0.05 Å. The bond lengths between surface atoms in the optimized 3B model are 2.38 Å for Ga1-Se1, 2.48 Å for Ga1-Se2, and 2.46 Å for Ga3-Se2, which are close to the bond length in bulk GaAs (2.45 Å) and $\hbox {Ga}_{2}\hbox {Se}_{3}$ (2.39 Å)^30^.

**Table 1:** Atomic coordinates in the optimized 3B model in Å. Atomic displacement in the *y* direction is not considered. The origin of the *z*-coordinate is at the outermost Ga layer of the bulk-terminated GaAs(001) surface. The values obtained from DFT calculations are normalized to the experimental lattice constant of GaAs (5.6538 Å).

Atom	DFT		LEED	
in Fig. 3b	*x*	*z*	*x*	*z*
Se1	$0.000^{*}$	$+1.217$	$0.000^{*}$	$+1.219$
Ga1	$0.000^{*}$	$-0.073$	$0.000^{*}$	$-0.075$
Se2	$+2.082$	$-1.421$	$+2.085$	$-1.241$
Ga2	$+1.975$	$-2.849$	$+2.020$	$-2.856$
As1	$0.000^{*}$	$-4.232$	$0.000^{*}$	$-4.217$
As2	$+3.998^{*}$	$-4.268$	$+3.998^{*}$	$-4.268$

* bulk value.

Figure 2f shows the Se 3d spectrum measured from the ($2\times 1$) surface. The spectrum is composed of two components denoted S1 (53.9 eV) and S2 (54.9 eV). While the two Se components with the same energy difference have been reported earlier^6,7,12,16,17^, two different interpretations have been made. Maeda et al.^7^ and Gonzarlez et al.^16^ have assigned the lower and higher binding-energy components to the Se atoms at the outermost layer and subsurface layers, respectively, which is contrary to the assignment reported in refs.^6^ and^12^.

In the 3B structure (Fig. 3b), the amount of Se1 and Se2 atoms are 0.5 ML and 1.0 ML, respectively, in good agreement with the intensity ratio of Se1/Se2 components ($\sim$0.5). Thus, it is likely that lower (higher) binding-energy component corresponds to the Se atoms at the outermost layer (third layer). The present peak assignment agrees with those discussed in refs.^7^ and^16^, and is consistent with the stabilization mechanism of the 3B model: as discussed earlier, the charge transfer from Se2 to Se1 occurs in the 3B structure, which causes the peak shifts of the Se 3d spectra of Se1 and Se2 atoms to lower and higher binding energies, respectively.

**Figure 5:**
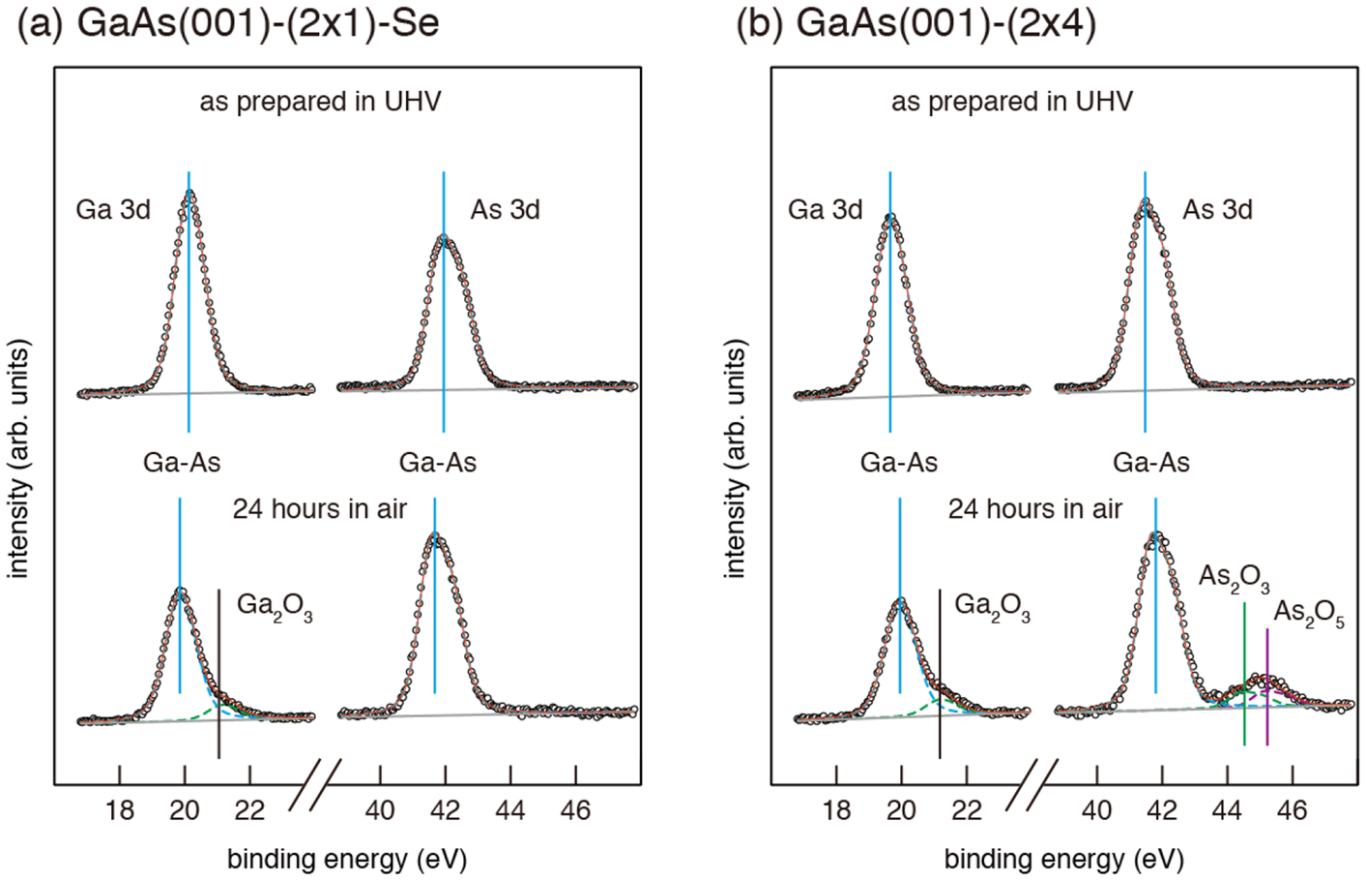
XPS spectra of Ga3d and As 3d measured from the Se-treated GaAs(001)-($2\times 1$) (**a**) and clean GaAs(001)-($2 \times 4$) (**b**) surfaces before and after the surface was exposed to air.

Next, we examined the chemical stability of the Se-treated ($2\times 1$) surface under ambient conditions. Figure 5 shows Ga 3d and As 3d XPS spectra measured before and after the Se-treated-($2\times 1$) surface (a) and the clean ($2 \times 4$) surface (b) were exposed to air for 24h. When the ($2 \times 4$) surface was exposed to air, additional components corresponding to $\hbox {As}_{{2}}\hbox {O}_{{3}}$, $\hbox {As}_{{2}}\hbox {O}_{{5}}$ and $\hbox {Ga}_{{2}}\hbox {O}_{{3}}$ phases were observed, and the sharp ($2 \times 4$) RHEED patterns changed to weak ($1 \times 1$) ones (Figure S2(b) in the Supplementary Material). On the other hand, the Se-treated surface show weak ($2\times 1$) RHEED patterns even after being exposed to air (see Figure S2a in the Supplementary Material). While $\hbox {Ga}_{{2}}\hbox {O}_{{3}}$ was formed also on the Se-treated surface, the formation of arsenic oxides was not confirmed. Early studies have shown that the surface states which pin the Fermi level are associated with arsenic oxides^31,32^. Since, as can be seen in Fig. 3b, the 3B model has no As atoms exposed to vacuum, it is likely that the atomic geometry is effective in suppressing the oxidation of As, leading to the improved electrical properties of GaAs-based devices.

The present results indicate that Ga oxides are preferentially formed, but the oxidation of Se and As is suppressed on the Se-treated ($2\times 1$) surface (see Figure S3 in the Supplementary Material for the result of Se). While the results are broadly consistent with those reported by Scimeca et al.^33^, their results showed the onset of the oxidation of Se and As for the shorter exposure time of 3h. Such a discrepancy could be ascribed to the quality of the Se-treated surface: the samples in ref.^33^ were prepared by exposing the GaAs(001) surface to the Se flux at a low temperature of $450^{\circ }\hbox {C}$. We confirmed that the ($2\times 1$) surface prepared at $450^{\circ }\hbox {C}$ is disordered and that the annealing at a higher temperature of $600^{\circ }\hbox {C}$ is necessary to form well-ordered ($2\times 1$) surface. Since disordered surfaces contain more defects, it is likely that the defects act as the sites for the oxidation reaction.

As mentioned earlier, DFT calculations have shown that the 6A and 1A structures are the most stable under more and less Se-rich conditions. To check the possible formation of structures other than the 3B model, such as 1A and 6A, the GaAs(001) surfaces were treated with Se under more Se-rich and Se-deficient conditions. The ($2\times 1$) surface with the 3B structure are formed by closing the Se shutter at temperatures ranging from 300 to $580\,^{\circ }\hbox {C}$ after the sample was annealed at $600^{\circ }\hbox {C}$ under the Se flux. To prepare Se-deficient surfaces, the sample was prepared by closing the Se shutter at a slightly higher temperature of $610^{\circ }\hbox {C}$. The surface showed weak 1/3-order reflections in the RHEED pattern taken along the [${\overline{1}}$10] direction (see Figures S4a and b, in the Supplementary Material). In the STM image of the ($2 \times 3$) surface (Fig. S4c in Supplementary Material), bright but discontinuous lines are observed along the [110] direction. Since the Se 3d photoemission intensity is one third of that from the ($2\times 1$)-Se surface, the ($2 \times 3$) surface has a Se-deficient structure. On the other hand, as can be seen in Fig. 1a and b, the 1A structure contains more As atoms in the surface layer as compared with the 3B structure. Since the annealing of the GaAs samples without supplying As molecules usually results in the As-deficient surfaces, the more As-rich 1A model is unlikely to be a structure component of the ($2 \times 3$) surface. This is supported by the LEED analysis: the LEED $I-V$ curves measured from the ($2 \times 3$) surface could not be reproduced by the 1A model. In addition, we confirmed that the 1A structure could not be formed by exposing the ($2\times 1$) and ($3 \times 2$) surfaces to As molecular beams.

The 6A structure is more Se-rich and As-deficient: as shown in Fig. 1c, the As atoms at the first, third and fifth layers are replaced by Se atoms. Thus, to promote the exchange reaction between Se and As for the formation of the 6A structure, the Se molecular beam cracked at $650^{\circ }\hbox {C}$ with a higher beam equivalent pressure of $2\times 10^{-7}$ Torr was used. After the sample was annealed at a higher temperature of $650^{\circ }\hbox {C}$ under the Se flux, the temperature was decreased to below $250^{\circ }\hbox {C}$ and the Se shutter was closed. The LEED and STM results obtained from the resultant surface are almost the same with those for the 3B structure. Thus, it is plausible that the amount of excess Se on the surface is not enough to form the Se-rich 6A structure under the present experimental conditions.

## Conclusions

We have studied the atomic structure of the Se-treated GaAs(001) surface. On the basis of DFT calculations and complementary experimental techniques of LEED, STM, and XPS, we found that the Se treated GaAs surface has the atomic structure consisting of two-fold coordinated Se atom at the outermost layer and the three-fold coordinated Se atom at the third layer. This atomic geometry is effective in suppressing the oxidation of As atoms in the surface layers of Se-treated GaAs(001).

### Supplementary Information

Supplementary Information.

## Data Availability

The data that support the findings of this study are available from the corresponding author upon reasonable request.
