# Supplementary material for: Atomic structure of the Se-passivated GaAs(001) surface revisited
Source: Sci Rep. 2023 Oct 24;13:18140. doi: 10.1038/s41598-023-45142-y (PMC10598220; doi:10.1038/s41598-023-45142-y)
Supplement: Supplementary file 1 — Supplementary Information. [file 41598_2023_45142_MOESM1_ESM.pdf]

Supplementary Materials

Atomic structure of the Se-passivated GaAs(001) surface revisited

Akihiro Ohtake[*,†], *Takayuki Suga*[‡], *Shunji Goto*[‡], *Daisuke Nakagawa*[‡], *and Jun Nakamura*[‡]

[†]*National Institute for Materials Science (NIMS), Tsukuba 305-0044, Japan*

[‡]*Department of Engineering Science, The University of Electro-Communications (UEC-Tokyo), Chofu, Tokyo 182-8585, Japan*

[*]**Corresponding Author:** OHTAKE.Akihiro@nims.go.jp

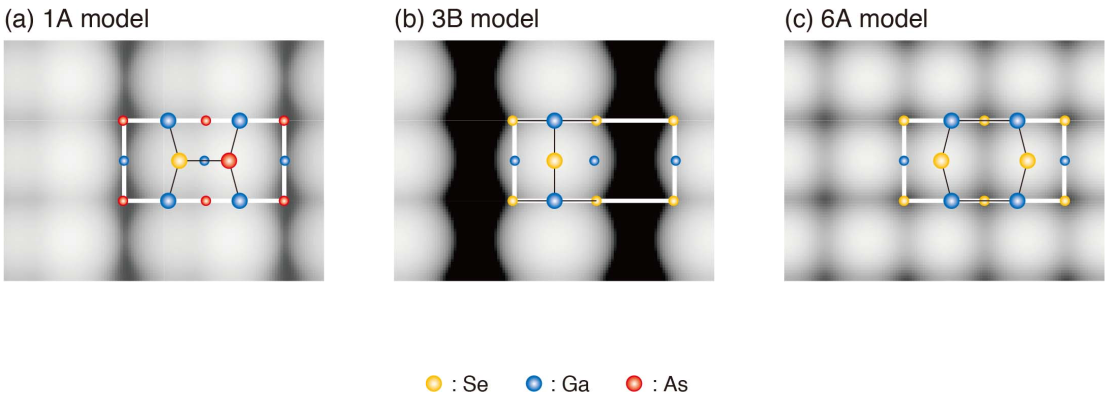

Figure S1: Simulated STM images for 1A (a), 3B (b), and 6A (c) models

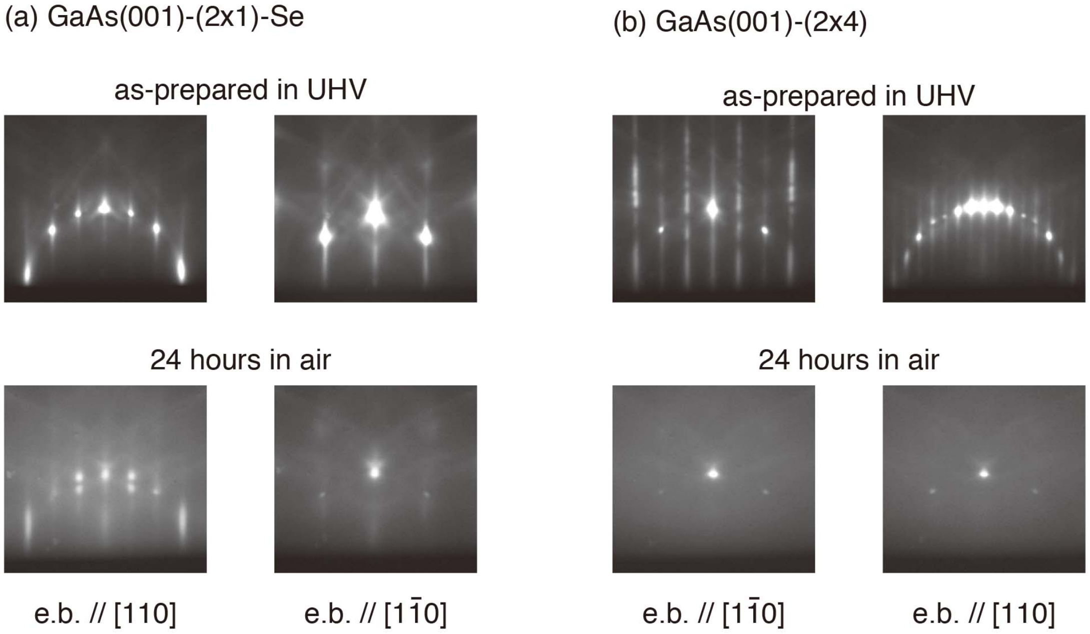

Figure S2: RHEED patterns taken from the Se-treated GaAs(001)-(2x1) (a) and clean GaAs(001)-(2x4) (b) surfaces before and after the surface was exposed to air.

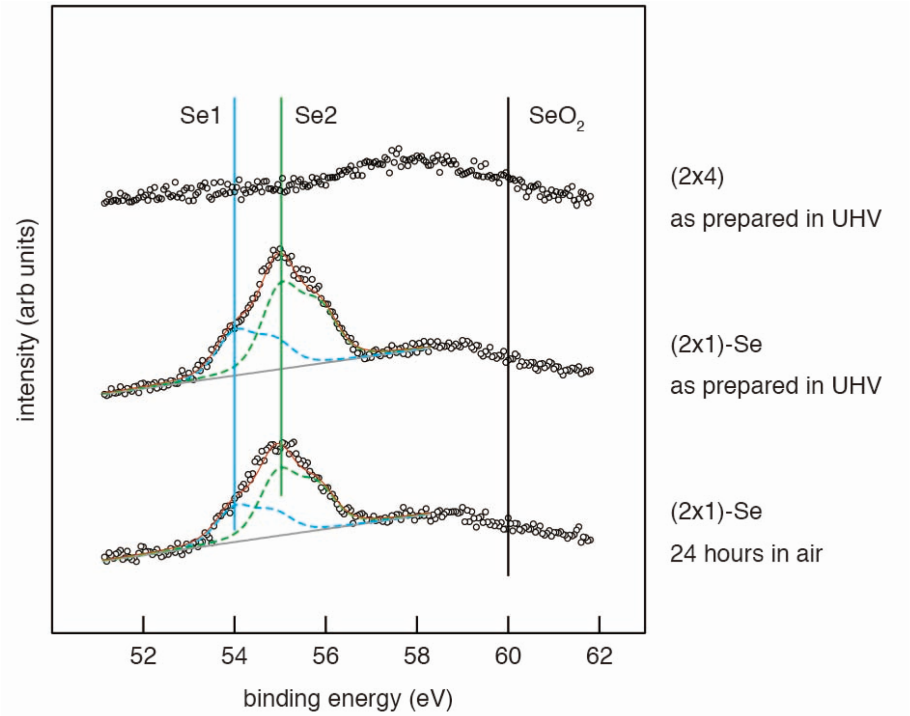

Figure S3: Se3d spectra measured from the Se-treated GaAs(001)-(2x1) surface before and after the surface was exposed to air. The spectrum measured from the GaAs(001)-(2x4) surface is also shown for comparison.

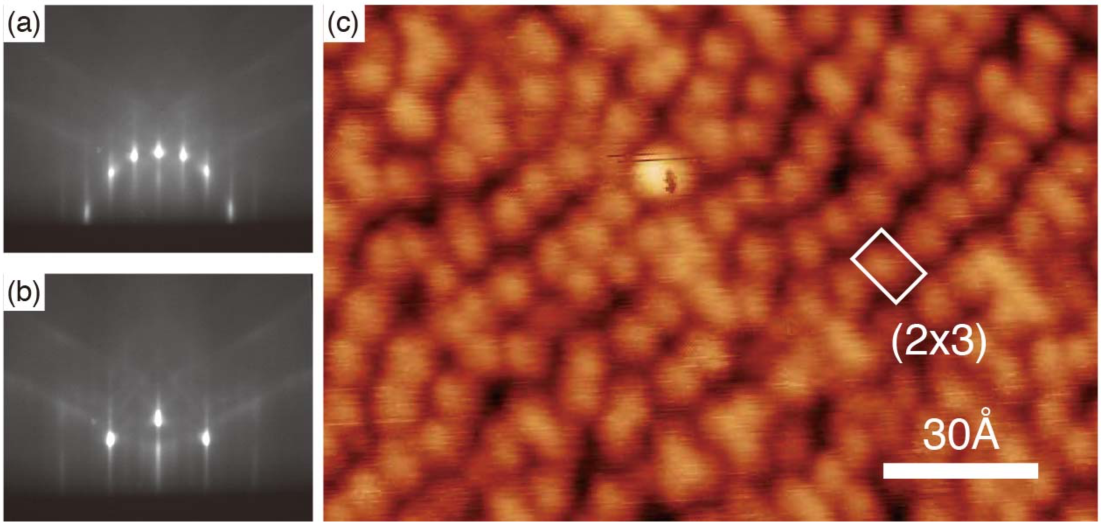

Figure S4: RHEED patterns of the Se-treated GaAs(001)-(2x3) surface taken along the [110] (a) and [1-10] (b) directions. (c) Typical filled-state STM image of the GaAs(001)-(2x3)-Se surface the image was taken with a sample bias of -3 V.
